# Photobleaching shapes the expression of plumage phenotypes

**DOI:** 10.1242/bio.062389

**Published:** 2026-01-14

**Authors:** Ismael Galván, Marta Araujo-Roque, Julene Gómez-Vicioso, Juan José Negro

**Affiliations:** ^1^ Department of Evolutionary Ecology, National Museum of Natural Sciences, CSIC, C/ José Gutiérrez Abascal 2, 28006 Madrid, Spain; ^2^ Department of Ecology and Evolution, Doñana Biological Station, CSIC, Avda. Américo Vespucio s/n, 41092 Sevilla, Spain

**Keywords:** Bird coloration, Birds of prey, Feather degradation, Pigmentation, Spanish imperial eagle, Sunlight exposure

## Abstract

Melanins are the most common pigments in animals and are known to experience bleaching (molecular degradation) under UV and visible radiations. However, melanin photobleaching effects on the appearance of animals under natural sunlight conditions are unclear. Here, we collected body feathers from developing Spanish imperial eagles *Aquila adalberti*, mainly pigmented by the orange pheomelanin, and monitored their reflectance properties during a 15-week sunlight exposure regime. Feather brightness significantly increased with exposure time following a power function, resulting in a 1.87-times increase in paleness and an obvious loss of feather integrity. Photobleaching thus explains the gradual increase in plumage paleness exhibited by juvenile imperial eagles, changing from dark orange to yellowish during the first months of age without the course of feather molt. Bleached plumage characterizes eagle immature phenotypes until reaching a contrasting blackish phenotype by progressive molt after 5-6 years, a period during which the feather degradation that accompanies bleaching may limit flight performance. Given the pheomelanin-pigmented plumages commonly observed in juvenile raptors, and in other groups of birds in which color disappears independently of molt (e.g. wheatears, genus *Oenanthe*), photobleaching arises as a source of phenotypic expression that may also drive life-history strategies such as crypsis and migration.

## Teaser

**Summary:** We show that sunlight affects plumage color in Spanish imperial eagles, presenting this factor as a source of phenotypic change in birds, and suggest it has a role influencing the evolution of life-histories.

## INTRODUCTION

Bird plumage coloration is not only one of the most diverse phenotypic traits when species are compared, but also a highly variable trait intraspecifically. Usually, plumage coloration varies with age (e.g. Galván and Moreno, 2009), and sometimes with gender. Starting with a pattern of downy feathers with a thermoregulatory function, the ontogeny of birds often makes plumage changes from a juvenile form to an adult-like structure in a period that can take up to several years and comprise of very distinct pre-adult plumages (Terrill and Schultz, 2023). While feather molt is the most common mechanism by which birds substitute pre-adult plumages (Terrill and Schultz, 2023), environmental agents can also induce such phenotypic change, but with an unknown importance. Solar radiation in particular is known to cause the fading of plumage color when produced by the deposition of carotenoid pigments on feathers (Blanco et al., 2005; Surmacki, 2008, 2011). This phenomenon is termed bleaching, and it remains unclear whether it produces plumage color shifts beyond seasonal annual changes, and whether it can affect pigments other than carotenoids.

Another class of pigments, namely melanins, are in fact the most common pigments in birds. The two chemical forms of melanin molecules, pheomelanin and eumelanin, are responsible for orange and black-brown hues, respectively (Galván and Solano, 2016). As melanins are synthesized only in melanocytes and there are normally no circulating melanins in the bloodstream, plumage color cannot be modified once melanins are deposited in the follicles during feather growth (Lin et al., 2013), in contrast to plant-derived carotenoids that can be incorporated in uropygial gland secretions and cosmetically applied onto the plumage to avoid bleaching (Chiale et al., 2021). This makes melanin-based plumage coloration particularly susceptible to bleaching until feathers are molted. Indeed, experimental treatments with both UV and visible radiation have been shown to modify the monomeric composition of natural melanins from melanocytes and hair and the absorption spectra of synthetic melanins in solution, indicative of photobleaching (Wakamatsu et al., 2012; Ito et al., 2018, 2019).

The consequences of photobleaching on the color properties (i.e. reflectance) of melanins are largely unknown, beyond absorbance effects that suggest color changes after exposure to sunlight of melanins from alpaca hair and *Escherichia colli* cells (Wakamatsu et al., 2021; Kim et al., 2026). Pigmented human hair has been reported to bleach (i.e. increase in brightness) after exposure to UV-visible light, but from artificial sources (Takahashi and Nakamura, 2004, 2005). Color properties (hue, saturation and brightness) of barn swallow *Hirundo rustica* feathers containing pheomelanin have been reported to increase after an experimental irradiation with UV light, but the effect has been ascribed to the pigment protoporphyrin IX, also present in barn swallow feathers (Hasegawa et al., 2024). The latter claim was based on associations between changes in color and pigment feather concentration, which makes it unclear because feather porphyrins photodegrade incomparably faster than melanins (Negro et al., 2009). Consequently, the potential effect of sunlight on animal colors produced by melanins remains untested.

Diurnal raptors (order Falconiformes) represent a good model to investigate the implications of photobleaching because their plumage is solely pigmented by melanins, except the presence of porphyrins in the *Elanus* kites (Negro et al., 2009), and usually exhibit pre-adult forms that are markedly distinct from definitive plumages (Ferguson-Lees and Christie, 2001). In some, particularly large species of diurnal raptors, the molt of feathers occurs gradually, following several cycles and molt waves, implying that definitive plumages are acquired only after years of consecutive pre-adult phenotypes (Zuberogoitia et al., 2018). Every molt cycle thus only affects a limited number of feathers, and the complete renewal of a given group of feathers (e.g. contour body feathers) may take several years (Zuberogoitia et al., 2018). This makes likely that photobleaching significantly contributes to shape pre-adult phenotypes. Due to their strict carnivorous diets, the juvenile (first) plumage of most diurnal raptors is predominantly pigmented by pheomelanin, probably due to the avoidance of the toxicity of excess cysteine during periods of low stress that pheomelanin synthesis favors (Rodríguez-Martínez and Galván, 2020). Juvenile raptors are thus richly orange colored, which conspicuously contrasts with the definitive plumage in some species (Rodríguez-Martínez and Galván, 2020). This is the case of true eagles (genus *Aquila*), large raptors in which orange juvenile plumages strongly contrast with the darker (caused by the pigment eumelanin) or unpigmented plumages of adults, with the sole exception of the golden eagle *Aquila chrysaetos* (Ferguson-Lees and Christie, 2001). Juvenile golden eagles, in fact, develop darker feathers with lower amounts of pheomelanin when reared in territories with higher exposure to solar UV-B radiation levels at a continental scale, which is probably an adaptive physiological response against damaging UV effects that eumelanin helps avoid (Galván et al., 2018). In other diurnal raptor, the griffon vulture *Gyps fulvus*, higher sunlight exposure times at a local scale have been associated to paler juvenile plumages, but climatization instead of bleaching has been adduced to explain the effect (Fargallo et al., 2018).

**Fig. 1.**
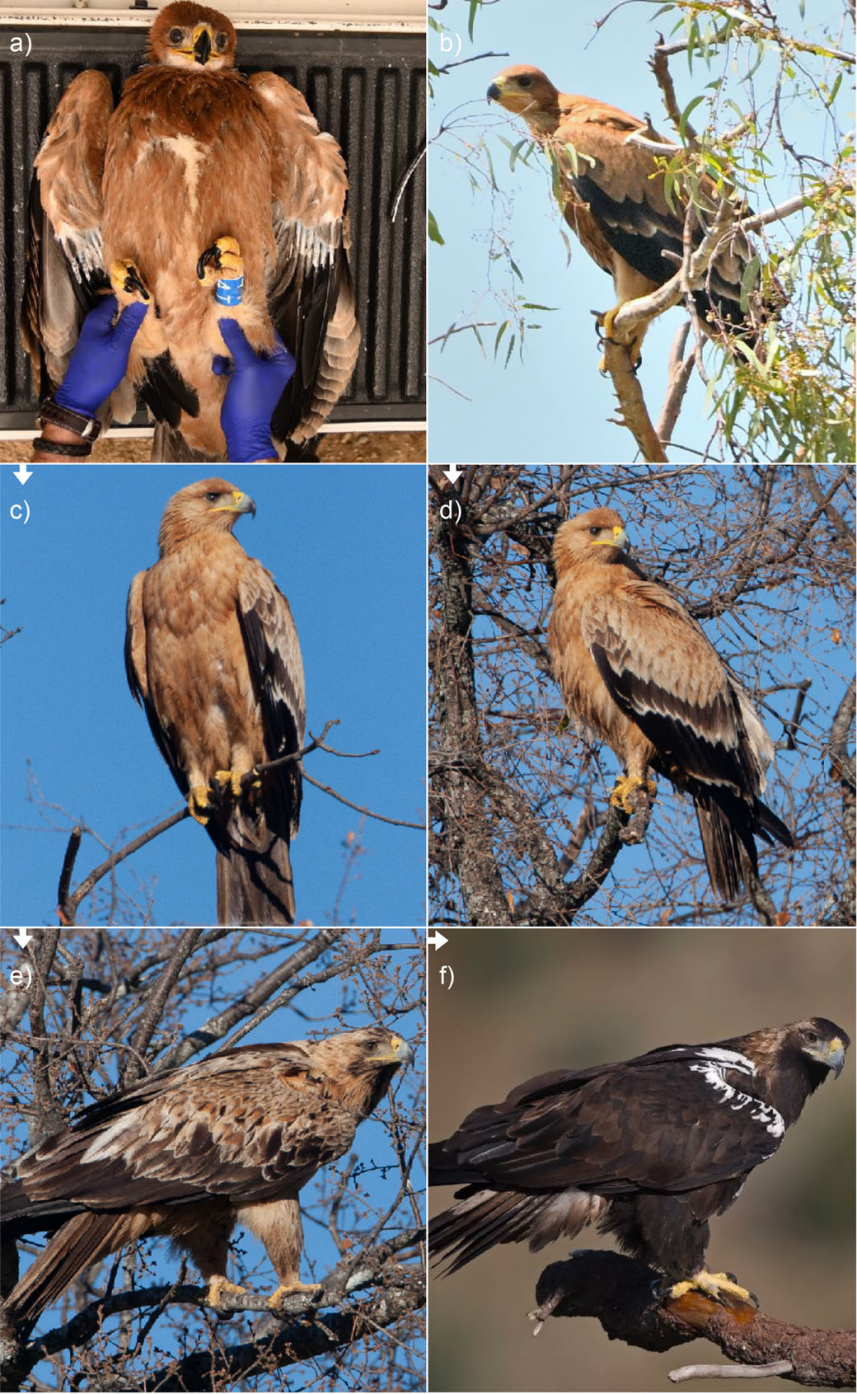
**Plumage phenotypes of Spanish imperial eagles of different ages.** (A,B) Juvenile (fledgling) eagles during handling (A) and in the surroundings of a nest (B), showing just developed dark orange plumage. (C,D) Juvenile eagles during the first week of January, at 7-8 months old, showing a markedly bleached, yellowish plumage. (E) Eagle during his third winter, showing a proportion of adult-like blackish feathers but still retaining juvenile bleached feathers. (F) >5-year-old eagle with fully developed adult-like phenotype acquired by molt. Arrows reflect the direction of age-related changes. Photo credits: A,B: Ismael Galván; C-E: Rafael Palomo; F: Clive Finlayson.

The Spanish imperial eagle *Aquila adalberti* is endemic to the Iberian Peninsula, the most threatened raptor in Europe and one of the rarest large eagles in the world (BirdLife International, 2021). It displays a definitive plumage almost entirely colored dark brown/black. This plumage form is not acquired until the fifth or sixth year of age and is the consequence of a gradual replacement of orange juvenile feathers by dark blackish feathers in loosely defined molting cycles (Sánchez et al., 2021; Fig. 1). The replacement of all juvenile feathers does not occur until the end of the fourth year, a period during which one annual molting cycle takes place, apparently covering only some contour body feathers (Sánchez et al., 2021). Retained juvenile feathers are perceived as a markedly pale orange color, and it is the proportion of pale body feathers that characterizes every pre-adult plumage phenotype (Sánchez et al., 2021). The fading of juvenile feathers is already perceived during the first few months of age (Fig. 1). Here, we experimentally test if the fading of juvenile body feathers in Spanish imperial eagles is due to bleaching by exposure to sunlight, with the aim of evaluating the potential contribution of photobleaching in determining the color phenotypes of birds pigmented by melanins.

## RESULTS

### Pigment composition of feathers

**Fig. 2.**
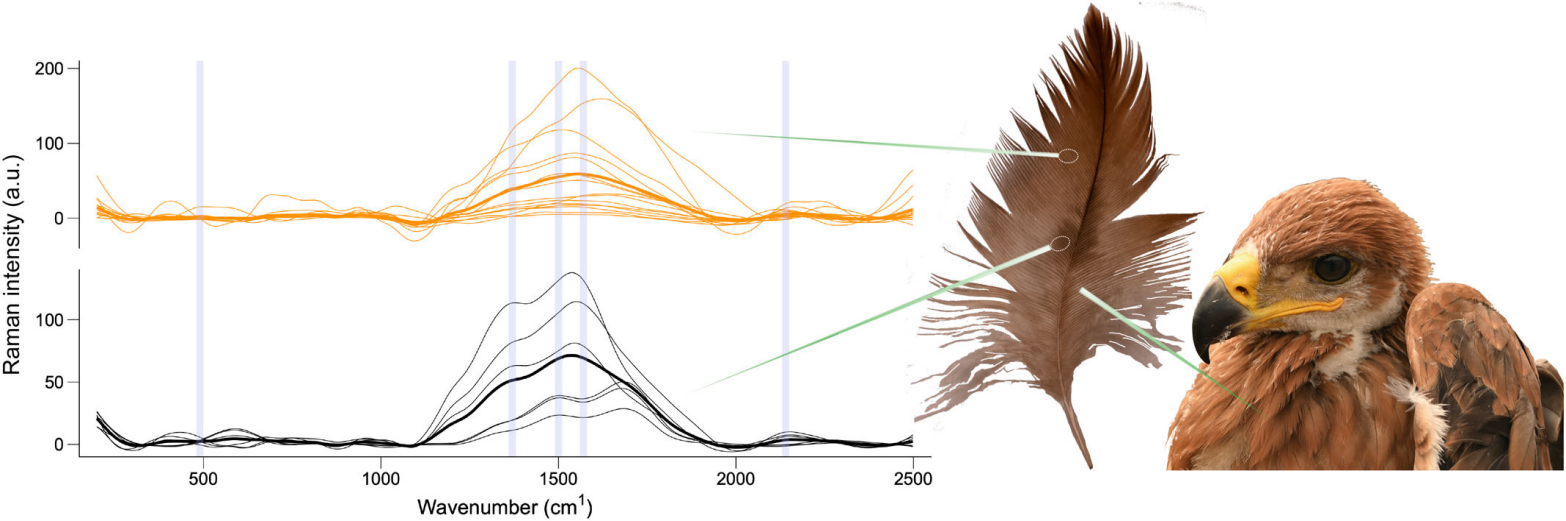
**Raman spectra of juvenile Spanish imperial eagle body feathers.** Measurements were taken of orange barbs and barbules, which gave rise to characteristic spectra of pheomelanin (orange curves), and at central dark brown areas (see feather image), which gave rise to characteristic spectra of eumelanin (black curves). Shaded blue areas indicate the position of diagnostic bands for pheomelanin (around 500, 1490 and 2000 cm^−1^) and eumelanin (around 500, 1380 and 1580 cm^−1^). Average pheomelanin and eumelanin Raman spectra are marked in bold.

The results of Raman spectroscopy analyses confirm that the orange color of feathers, which comprises most part of the juvenile plumage of Iberian imperial eagles (Fig. 1A-D), is caused by the pigment pheomelanin, while the dark brown color area that some feathers show around the rachis is caused by the pigment eumelanin (Fig. 2). However, some of the Raman spectra obtained from the dark brown area of feathers also show diagnostic Raman bands of pheomelanin, suggesting a mixed composition of both pheomelanin and eumelanin in these areas (Fig. 2). Similar to the fledgling feathers of a close species, the golden eagle (Galván et al., 2018), this mixture of pigments probably causes the shift found in the diagnostic Raman bands respective to the expected wavenumbers (Fig. 2).

### Sunlight effect on feather brightness

Wild juvenile Spanish imperial eagles show a significantly paler plumage coloration in the first winter of their life, exhibiting a markedly yellowish appearance as compared to the recently developed plumage in the nest (Fig. 1A-D). As only some Spanish imperial eagles molt their contour body feathers during their first winter [and always only some of these feathers (Sánchez et al., 2021)], it can be stated that such change in plumage coloration is due to bleaching and not feather replacement with molt. In subsequent years, before acquiring the definitive adult-like plumage, the body feathers that Spanish imperial eagles retain from previous years are yellowish (Fig. 1E), suggesting that bleaching characterizes the color phenotypes that these birds sport until their fifth year.

**Fig. 3.**
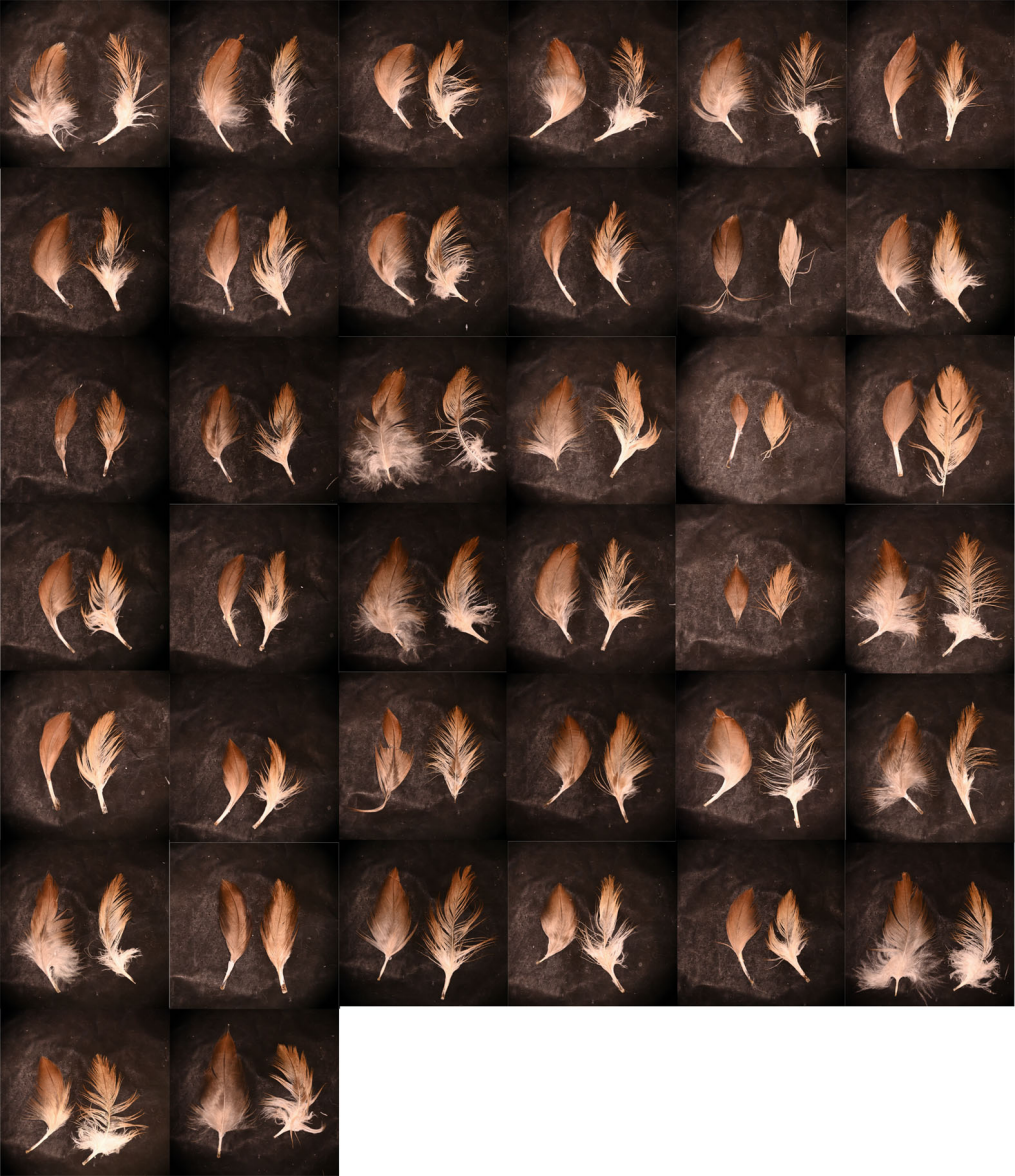
**Images of the juvenile Spanish imperial eagle dorsal body feathers used in the study.** Each pair of feathers corresponds to a different fledgling. In each pair, the feather on the left is the feather that was not exposed to sunlight, and the feather on the right was experimentally exposed to sunlight for 15 weeks. Photographs were taken under standardized lighting conditions and were not subjected to any editions of color.

**Fig. 4.**
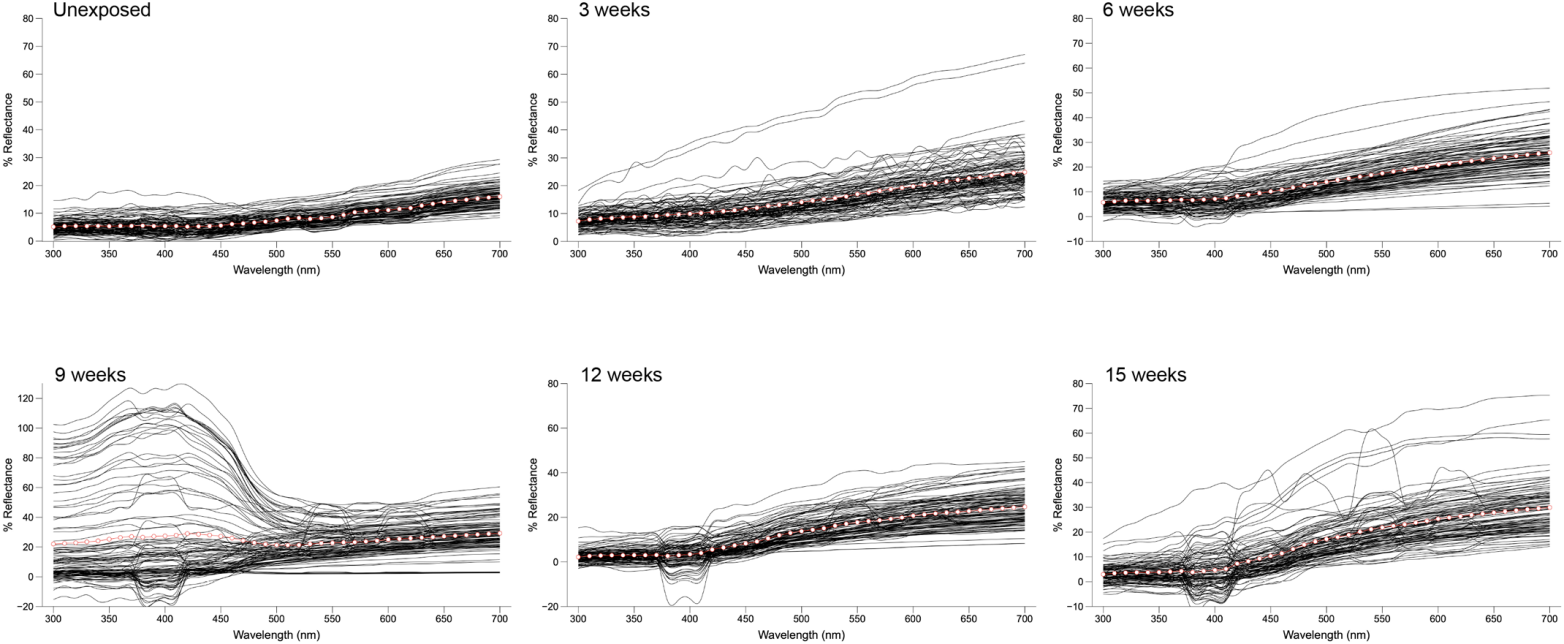
**Variation in reflectance spectra of juvenile Spanish imperial eagle body feathers during the sunlight exposure regime.** Each curve represents the average spectra of six readings obtained from a single feather. Total mean spectra are depicted as points along a red curve.

Color bleaching was confirmed in the feathers experimentally exposed to sunlight, as paling was already visually apparent in the first 3 weeks of exposure. At the end of the experiment, after 15 weeks of exposure, the color of feathers had paled considerably, and a loss of barbs/barbules and feather integrity was also apparent (Fig. 3). This color change was also evident in the reflectance spectra of feathers, which increased in values with exposure time (Fig. 4). A comparison of the mean initial brightness of feathers (mean±SE; 353.27±10.38%) with the mean brightness at the end of the experiment (659.25±31.24%) resulted in a mean increment of 86.6%, meaning that bleaching made the brightness of feathers increase 1.87-times after direct exposure to sunlight (Fig. 3).

**Fig. 5.**
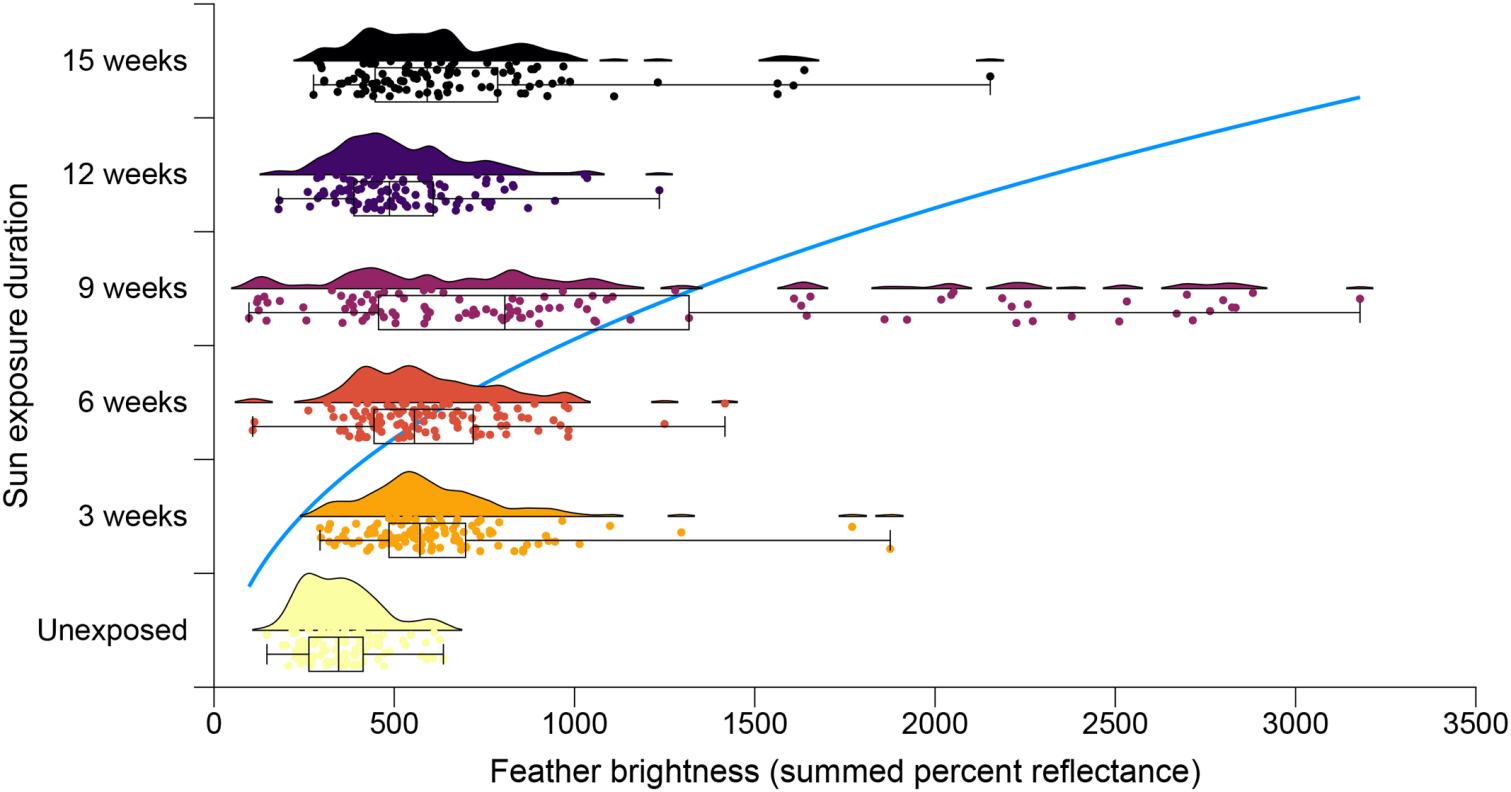
**Quantification of the bleaching effect on Spanish imperial eagle body feathers.** The color effect is quantified by the brightness of feathers, measured every 3 weeks over a period of 15 weeks, and represented by raincloud plots showing the distribution of data. Boxplots show medians and interquartile and min/max ranges. The blue curve describes the power function fitted to 629 measurements of brightness (*exposure duration*=0.217×*brightness*^0.405^).

The repeated measures analysis of feather brightness resulted in a final model that included highly significant effects of exposure time (*F*_1,590.44_=26.09, *P*<0.0001) and pigment identity (*F*_1,514.84_=4.06, *P*=0.044). This indicates that the brightness of feathers changed across the time of sunlight exposure (Fig. 5), and that orange and dark brown areas of feathers differed in brightness, as expected. This effect was independent of sex and pigment identity, as the interaction of these terms with time was not significant (sex × time: *F*_1,449.64_=0.18, *P*=0.670; pigment × time: *F*_1,589.27_=0.91, *P*=0.341). The effect of sex alone was also not significant (*F*_1,39.37_=0.10, *P*=0.752), indicating an absence of sexual dichromatism in Spanish imperial eagles already in their first plumage.

Feather brightness changed with sunlight exposure tending to an asymptote (Fig. 5). This was as expected, because the color perceived in the feathers at the end of the experiment suggested that it was close to a maximum level of bleaching (Fig. 3), and because an intermediate and reversible darkening (i.e. increase in reflectance after 9 weeks of sunlight exposure in our case) has in fact been described during the photobleaching of synthetic melanins in solution, regarding absorbance (Wakamatsu et al., 2012). Consequently, the change in brightness with exposure time of Spanish imperial eagle feathers fitted better a power function (*brightness*=*a*×*time*^*b*^) than a linear function (*brightness*=*a*+*b*×*time*) (ΔAIC=12.44) or an exponential function (*brightness*=*a*×*b*^*time*^) (ΔAIC=15.75) (Fig. 5). Both coefficients of the power function were highly significant (a=468.86, t=14.54, *P*<0.0001; b=0.27, t=5.29, *P*<0.0001; d.f.=627).

## DISCUSSION

In June 1883, the British naturalists Abel Chapman and Walter J. Buck examined an eagle from Jerez de la Frontera in southwestern Spain, originating from what is now Doñana National Park, whose plumage, described as tawny chestnut, was so pale that it prevented them from identifying the species (Chapman and Buck, 1893). The eagle specimen, which was categorized as an undetermined species in The Great North Museum of Newcastle (catalog number NEWHM: 2000.H1287), was examined by four other reputed ornithologists of their time, who were equally unable to determine its specific status, giving us an idea of how prevalent the pale plumage coloration of this eagle was (Fig. S1A). Upon direct examination of the specimen by one of the authors of this paper (J.J.N.) in the museum, we can state that it is a 2.5-year-old Spanish imperial eagle. It was not an isolated case, as similarly extremely pale sub-adult eagles have been observed during the winter in Doñana in the past (Chapman and Buck, 1893).

In fact, one of the fledglings included in our study, from a nest in Doñana National Park, exhibited the same extremely bleached phenotype described by Chapman and Buck (1893) (Fig. S1B-D). Doñana mostly comprises of a marsh habitat almost devoid of trees (our sampled fledgling's nest was in an isolated blue gum *Eucalyptus globulus*), considerably different to the typical habitat of Spanish imperial eagles in most of their distribution range (dense or sparsely forested Mediterranean forest; Ferrer, 2001), and is among the areas receiving highest levels of solar irradiance in Spain (Moreno et al., 2011). Our results show that photobleaching shapes the appearance of sub-adult Spanish imperial eagles, making them pale from the first weeks of life, which can generate extremely bleached phenotypes in areas highly exposed to sunlight such as Doñana. Bleaching is particularly evident in their first winter when eagles are about 7-8 months old (Fig. 1C,D), giving rise to a phenotype that even has a specific word in Spanish (‘pajizo’, or straw-colored), but it probably persists until the eagles acquire their definitive plumage in their fifth or sixth year (Fig. 1E,F).

As far as we know, this is the first report of color consequences of photobleaching by sunlight exposure in a tegumentary tissue pigmented by melanins. Beyond a color/aesthetic consequence of sunlight exposure, the photobleaching of plumage may have relevant evolutionary implications as it could affect the performance of flight and also concealment and, thus, crypsis. Camouflage is also crucial to apical predators such as eagles and owls, in order to minimize early detection by their prey (Negro et al., 2025). While this will have to be investigated in the future, studies on wool and human hair have shown that photobleaching is accompanied by a structural degradation of melanin granules and of the amino acids constituent of keratin fibers on which melanin pigments are embedded (Takahashi and Nakamura, 2004, 2005; Millington, 2006; Nogueira et al., 2007). We found a significant deterioration of Spanish imperial eagle feathers at the end of the sunlight exposure period, with a relevant loss of mainly barbules, which prevented feather cohesion (Fig. 3). Thus, photobleaching of eagle feathers probably diminishes the mechanical protection that melanins provide (Schreiber et al., 2006), as well as flight performance (Sullivan et al., 2019). Our experiment does not fully mimic natural conditions, as birds can actively avoid direct sun exposure and also apply uropygial gland secretions onto the plumage, which may protect feathers from degradation (Vincze et al., 2013). Our estimated bleaching rate rather reflects a maximum level that can be reached by sun exposure.

Although pigment identity did not influence the sunlight exposure effect on the brightness of Spanish eagle feathers, we cannot completely rule out the possibility that pheomelanin is particularly susceptible to photobleaching, because pheomelanin and eumelanin were mixed in the dark brown areas of feathers, and only some fledglings had these color areas in their feathers. In red human hair, which is mainly pigmented by pheomelanin, this pigment has actually been reported as more susceptible to photobleaching than eumelanin (Takahashi and Nakamura, 2005). Pheomelanin-pigmented (orange) juvenile plumage has evolved in strict carnivorous birds, such as raptors, probably as an adaptive physiological strategy to avoid the toxicity that can cause an excess dietary cysteine (which is used to build the pigment) during development periods of low stress (Rodríguez-Martínez and Galván, 2020). After fledging, immature birds may thus pay the mechanical cost of bearing pheomelanin-pigmented plumage in terms of potential lack of protection and impaired flight, especially in large species such as eagles, in which the complete replacement of juvenile feathers takes years. This consideration may be of conservation value for the highly threatened Spanish imperial eagle, as there are an increasing number of juveniles that cross the Strait of Gibraltar (Morandini et al., 2020). However, there is no settlement success of juvenile Spanish imperial eagles in North Africa, a territory from where the species disappeared in the 1960s, most likely because of an insufficient number of eagles making such migrations from Spain (Morandini et al., 2020). As feather degradation impairs sustained flight (Sullivan et al., 2019), and crossing water barriers such as the Strait of Gibraltar entails high energetic costs for soaring raptors, with juveniles showing a particularly low crossing performance (Santos et al., 2020), the strategy to facilitate the re-colonization of North Africa by immature Spanish imperial eagles should be revisited.

The ecological and evolutionary implications of our findings are not only relevant for the Spanish imperial eagle, however. First, the juvenile plumage of other true eagles seems to experience the same bleaching effect, as Ellis and Lish (2006) reported the results of exposing golden eagle secondary remiges to sunlight, verbally describing color bleaching after virtually the same exposure time (106 days) that we considered in our experiment. Observational evidence also indicates that bleaching significantly determines the expression of the plumage phenotype in raptors from orders different from Accipitriformes, such as falcons (order Falconiformes). The case of the lesser kestrel *Falco naumanni* is also notable, being one of the few sexually dichromatic raptors (Ferguson-Lees and Christie, 2001) and shows a clear pheomelanin-based color bleaching effect in back feathers (Fig. S2), raising the question of whether bleaching may exert an influence on sexual selection.

Beyond raptors, the bleaching of plumage may affect life-history strategies in other groups of birds. This is the case in wheatears (genus *Oenanthe*), with several species showing orange coloration that is visible only shortly after the annual molt in the summer, and disappears or becomes extremely pale in the distinctive breeding plumage displayed in the spring without the course of molt (van Duivendijk, 2024) (Fig. S1E,F). This effect is assumed to be due to feather wear (van Duivendijk, 2024), and as the orange color is caused by the pigment pheomelanin (I.G., unpublished data), it is likely that wearing results from bleaching. Photobleaching, therefore, seems to shape the breeding plumage phenotype of wheatears, potentially driven by the higher susceptibility of pheomelanin to such an effect (see above). Recently, throat and mantle coloration among wheatear species, regarding black-or-white variability, has been associated with variation at the gene that codes for the agouti-signaling protein (*ASIP*) in melanocytes (Lutgen et al., 2025). Interestingly, however, ASIP modifies intra-melanocytic cAMP levels, affecting the activity of the central enzyme in the melanogenesis pathway (tyrosinase) in a way that favors pheomelanin production in birds (Nadeau et al., 2008; Rodríguez-Martínez et al., 2019). While Lutgen et al. (2025) categorized the phenotype of wheatears apparently only considering the breeding plumage, understanding that *ASIP* controls the production of a pigment that is bleached and disappears may allow obtaining a more comprehensive view of phenotypic evolution in these birds. Larks (family Alaudidae) represent another group of birds with pheomelanin-based coloration that show a rapid degradation of plumage after a single annual molt, probably adaptively as it may contribute to camouflage (Negro et al., 2019) and is likely derived from bleaching. With no intention of being exhaustive, passerines in other families with a single annual molt, such as the snow bunting *Plectrophenax nivalis* (family Emberizidae), also show a conspicuous seasonal plumage color change consisting in the loss of pheomelanin-based orange coloration (van Duivendijk, 2024). These examples suggest that photobleaching of pheomelanin-based pigmentation is a widespread mechanism for the acquisition of color phenotypes, particularly those characteristics of the breeding season, among birds.

Another implication of the bleaching of pheomelanin-pigmented plumage may be related to the evolution of migratory habits. Given the significant melanin contribution to the mass of feathers recently reported (Galván and Gómez-Vicioso, 2025), migration may be facilitated in those species that get rid of pheomelanin by bleaching and thus produce lighter plumage.

In conclusion, we show that natural conditions of sunlight exposure induce the color bleaching of juvenile Spanish imperial eagle feathers, almost doubling their brightness/paleness after 15 weeks of exposure. This indicates that photobleaching shapes the phenotype of Spanish imperial eagles, and probably other raptors as well, until acquiring their definitive, darker plumage by molt after 5-6 years. During that period, the degradation of feathers that accompanies bleaching is likely to constrain life-history strategies related to flight performance such as migration. In species pigmented by a combination of melanin forms, the photobleaching of pheomelanin may have evolved, however, as an adaptive strategy that reduces plumage weight.

## MATERIALS AND METHODS

### Bird sampling

During the course of a study on conservation physiology, we visited 17 active nests of Spanish imperial eagles in Andalucía, southern Spain, in May-July 2023 and 2024. Visits to nests took place when the fledgling eagles were between 40 and 60 days old and their juvenile plumage was almost fully developed (Fig. 1A,B). The fledglings were lowered to the base of trees where the nests were located, where we marked them with numbered rings, took morphological measurements (tarsus and forearm length, and body mass), and plucked three contour body feathers from the back (from one fledgling we could obtain only two feathers). We put the feathers in plastic bags and stored them in dark conditions until the analyses were made. We obtained feathers from a total of 39 fledgling Spanish imperial eagles. The fledglings were released back to the nests after taking samples and measurements.

The age and sex of fledglings were determined on the basis of plumage characteristics and forearm length, respectively (Ferrer and de le Court, 1992), although the sex was later confirmed by PCR analyses made on blood samples taken from a brachial vein and using the primers E6 (2550F) and E7 (2718R) to amplify the genes *CHD1-Z* and *CHD1-W* (Griffiths et al., 1998; Fridolfsson and Ellegren, 1999). Molecular sex determination could be done in all but three fledglings, but morphological measurements correctly predicted sex in 32 out of 38 cases (84%) for which both measurements were available, thus we used morphological measurements to sex the three fledglings without molecular sex determination.

### Experimental design

Two out of the three feathers collected from each fledgling eagle were mounted on a whiteboard and placed horizontally outdoors, allowing exposure to direct sunlight by avoiding shadows from any element of the surroundings. The experiment took place in Navacerrada, central Spain, within the natural distribution range of the species (Ferrer, 2001), between June and September 2025. These conditions therefore allowed us to consider the sunlight period and intensity that Spanish imperial eagles naturally experience during their first weeks of life.

Before exposing the feathers to sunlight, we obtained the reflectance spectra of the feathers to measure their original color expression (see below). After exposing the feathers, we obtained their reflectance spectra every 3 weeks, until conducting a total of five measurements 15 weeks after exposure. Parallel to reflectance measurements, we photographed the feathers to illustrate the progress of bleaching. Photographs were made with a Nikon D6 digital camera (Nikon Corporation, Tokyo, Japan) under standardized lighting conditions by using a Godox (Shenzhen, China) Ring48 ring flash to uniformly illuminate the samples.

### Raman spectroscopy

To characterize the melanin forms responsible for plumage colors of Spanish imperial eagles, we analyzed feathers that had not been exposed to sunlight from five fledglings from different nests by micro-Raman spectroscopy, as pheomelanin and eumelanin exhibit distinctive Raman signals that can be used to non-invasively identify and quantify them (Galván et al., 2013). We used a Thermo Fisher DXR confocal dispersive Raman microscope (Thermo Fisher Scientific, Madison, WI, USA) with a point-and-shoot Raman capability of 1 μm spatial resolution and using a near-infrared excitation laser of 780 nm. For the orange pigmented area of feathers, laser power was set at 14 mW, integration time at 3 s, and number of accumulations at 20. Some feathers present a darker brown area around the rachis, for which we also obtained Raman spectra (Fig. 2). For these dark brown areas, laser power was set at 2 mW, integration time at 3 s, and number of accumulations at 13. All spectra were obtained using a 50× confocal objective and a slit aperture of 25 μm. The system was operated with Thermo Fisher OMNIC 8.1 software. Calibration and alignment of the spectrograph were checked using pure polystyrene.

The laser beam was focused on different barbs and barbules until three single spectra of melanins for each feather and area were obtained. The two melanin forms show distinctive Raman spectra comprising three diagnostic bands each, located at about 500, 1500 and 2000 cm^−1^ in the case of pheomelanin and at about 500, 1380 and 1580 cm^−1^ in the case of eumelanin. These appear as two clearly distinguishable spectral shapes; thus, a single Raman spectrum can usually be assigned to either pheomelanin or eumelanin (Galván et al., 2013). In the case of Spanish imperial eagle feathers, however, we found that Raman signals from pheomelanin and eumelanin appear mixed in some spectra, which show the diagnostic bands of both melanin forms with some band displacements (Fig. 2).

### Reflectance spectrophotometry

We analyzed the feathers by UV-visual reflectance spectrophotometry to assess color expression. An Ocean Optics (Duiven, The Netherlands) Jaz spectrophotometer (range: 220-1000 nm) with UV (deuterium) and visible (tungsten-halogen) lamps and a bifurcated 400 μm fiber optic probe (Ocean Optics) was used. The fiber optic probe both provided illumination and received light reflected from the sample, with a reading area of ∼1 mm^2^. We mounted the feathers on a light-absorbing foil sheet (Metal Velvet coating, Edmund Optics, Barrington, NJ, USA) to avoid any background reflectance. Measurements were taken at a 90° angle to the sample. All measurements were relative to a diffuse reflectance standard tablet (WS-1, Ocean Optics), and reference measurements were frequently made. Six readings on different points of the orange pigmented portion of feathers, and on the dark brown areas surrounding the rachis, was obtained for each feather, removing the probe after each measurement. Reflectance curves were determined by calculating the median of percentage reflectance in 10-nm intervals. We used total brightness, defined as the summed reflectance across the 300-700 nm range, as a measure of color expression of feathers. The term paleness is here used to refer to the change in color perception that results from an increase in brightness.

### Statistical analyses

We run a linear mixed model with a repeated measures design to test if time of exposure to sunlight affected the brightness of feathers. In this model, fledgling identity was a random factor, the sex of fledglings was a between-subjects factor, and exposure time was a within-subjects factor. As the pigment composition of orange and dark-brown areas of feathers differed (see Raman spectroscopy results below), we also included pigment identity (pheomelanin versus eumelanin) as a between-subjects factor in the model to control for this potential source of variation in feather brightness. The model included two-way interactions between factors, and we followed a stepwise removal of non-significant terms. We used the lmerTest R package (Kuznetsova et al., 2017) to conduct these analyses. As we were also interested in how feather brightness changed with exposure time, we compared the linear fitting of brightness against time with non-linear fitting functions (exponential and power), using the Akaike information criterion (AIC) to compare models. We used the minpack.lm R package (Elzhov et al., 2023) to fit the non-linear function and test for the significance of regression coefficients.

## Supplementary Material

10.1242/biolopen.062389_sup1 Supplementary information
