# Supplementary material for: Photobleaching shapes the expression of plumage phenotypes
Source: Biol Open. 2026 Jan 14;15(1):bio062389. doi: 10.1242/bio.062389 (PMC12833804; doi:10.1242/bio.062389)
Supplement: Supplementary information [file biolopen-15-062389-s1.pdf]

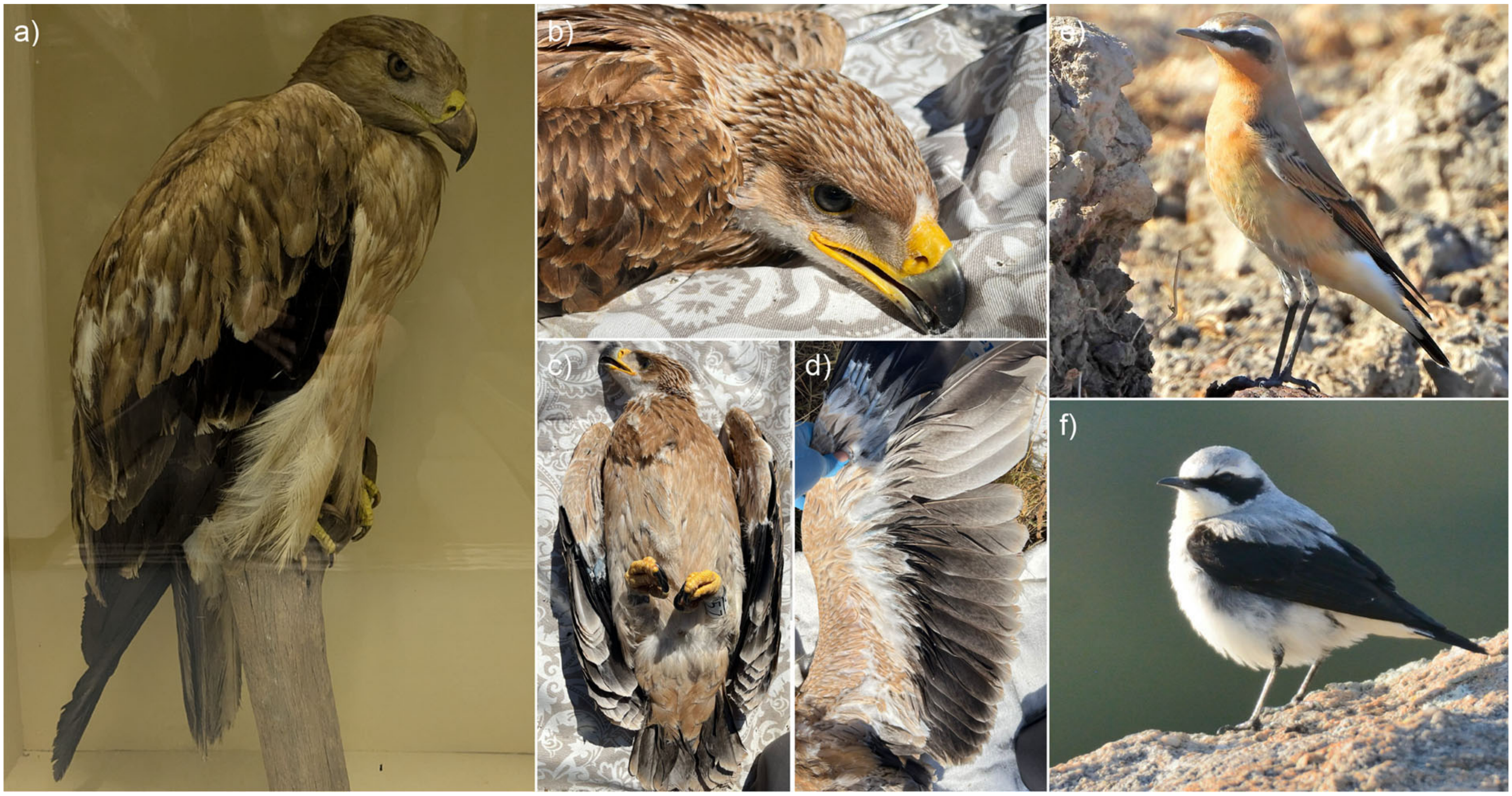

Fig. S1. Images of extremely bleached plumage phenotypes. a: Undetermined juvenile eagle specimen from Doñana collected in 1885 and kept in The Great North Museum of Newcastle (catalog number NEWHM : 2000.H1287). b-d: Juvenile Spanish imperial eagle sampled in Doñana for the present study, showing extremely pale plumage coloration similar to that observed in a). e-f: Adult male Northern wheatear *Oenanthe oenanthe* in September (e), with recently molted orange plumage pigmented by pheomelanin, and in June (f), with complete absence of orange color previous to molt. Photo credits: a: Dan Gordon; b-d and f: Ismael Galván; e: Juan José Negro.

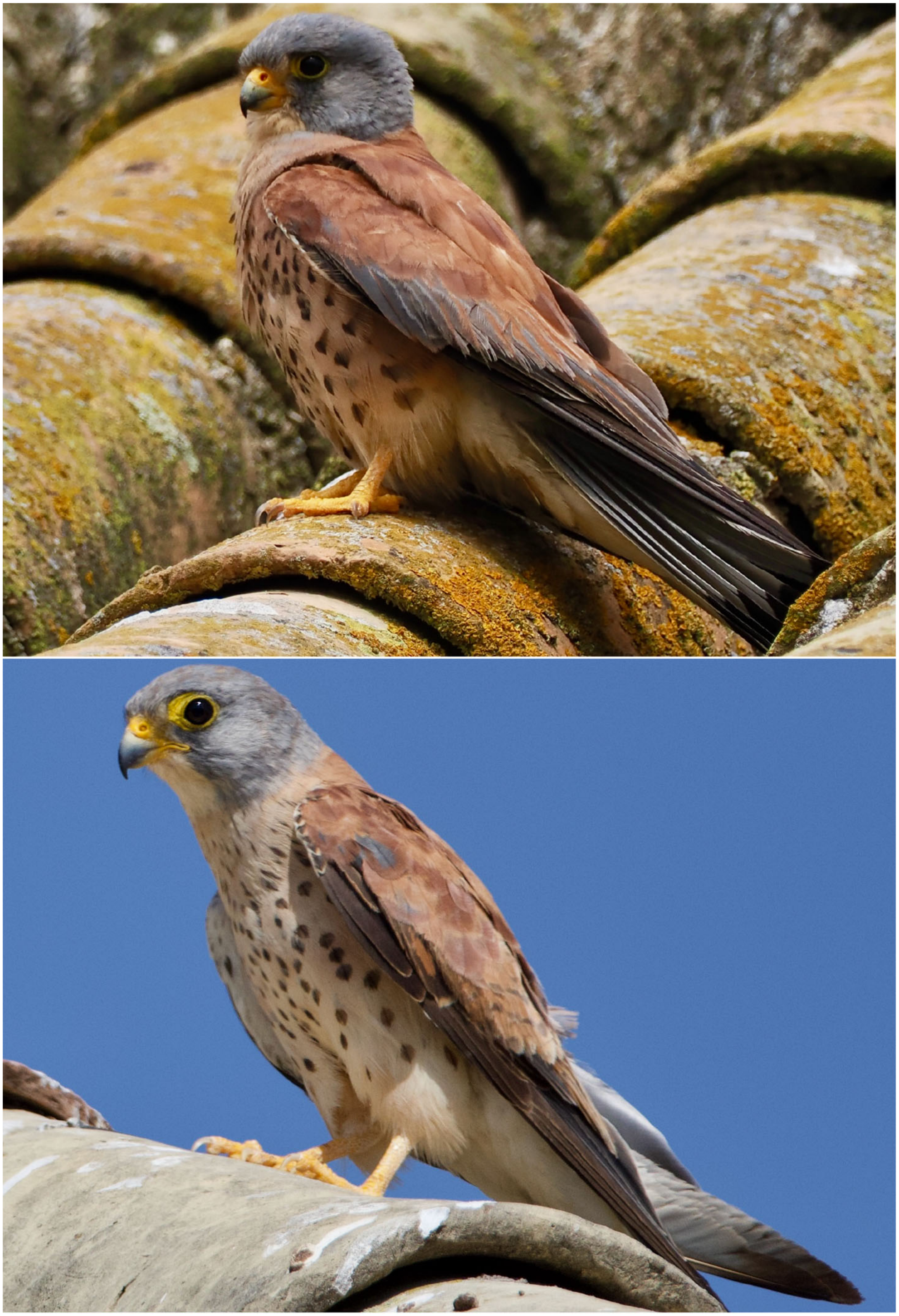

Fig. S2. Images of adult male lesser kestrels. The birds show bleached dorsal feathers that contrast with darker, recently molted feathers. Photo credits: Juan José Negro.
